# G protein γ subunit 7 induces autophagy and inhibits cell division

**DOI:** 10.18632/oncotarget.8559

**Published:** 2016-04-02

**Authors:** Juanjuan Liu, Xinmiao Ji, Zhiyuan Li, Xingxing Yang, Wenchao Wang, Xin Zhang

**Affiliations:** ^1^ High Magnetic Field Laboratory, Chinese Academy of Sciences, Hefei, Anhui, 230031, P. R. China; ^2^ University of Science and Technology of China, Hefei, Anhui, 230036, P. R. China

**Keywords:** GNG7 (G protein γ subunit 7), autophagy, cell death, cell division, actin

## Abstract

GNG7 (G protein γ subunit 7), a subunit of heterotrimeric G protein, is ubiquitously expressed in multiple tissues but is down-regulated in various cancers. Its expression could reduce tumor volume in mice but the mechanism was not clear. Here we show that GNG7 overexpression inhibits cell proliferation and increases cell death. GNG7 level is cell cycle-dependent and it regulates actin cytoskeleton and cell division. In addition, GNG7 is an autophagy inducer, which is the first reported Gγ protein involved in autophagy. GNG7 knockdown reduces Rapamycin and starvation-induced autophagy. Further analysis reveals that GNG7 inhibits MTOR in cells, a central regulator for autophagy and cell proliferation. In conclusion, GNG7 inhibits MTOR pathway to induce autophagy and cell death, inhibits cell division by regulating actin cytoskeleton. These combined effects lead to the antitumor capacity of GNG7.

## INTRODUCTION

Heterotrimeric G proteins are activated by upstream receptors and their specific interactions are the major factors governing the differential functions of the large numbers of GPCRs (G protein coupled receptors). Humans have 23 Gα (including Gα_s_, Gα_i_, Gα_q/11_ and Gα_12/13_), 5 Gβ, and 12 Gγ subunits, as well as some regulators. In the traditional model of GPCR activation, Gα dissociates from Gβγ and initiates a signaling cascade in the cytoplasm. However, although G proteins are the major effectors of GPCRs at the cell membrane, mounting evidences suggest that G proteins have additional functions other than the traditional GPCR binding partners. For example, some Gα and Gβ proteins have been shown to be involved in cell division [1–8] and cytoskeletal actin and microtubules were shown to be regulated by some Gα and Gβ proteins [9–13]. In comparison with Gα and Gβ proteins, Gγ proteins are much less studied. Although it has been well established that the Gβγ works as a dimer, some reports show that Gβ or Gγ subunits interact with a number of novel binding partners having special domains and are functionally distinct from conventional Gβγ dimers [14–16], indicating that they have independent roles.

G protein γ subunit 7 (GNG7) is the first Gγ protein that was studied using a gene-targeting strategy in mice. GNG7 is a component of a Gα_olf_ protein that is responsible for A_2A_ adenosine or D1 dopamine receptor-induced neuro-protective response [17]. GNG7 is enriched in striatum and forms a heterotrimeric complex with Gα_olf_/Gβ_2_, which is coupled to D1 receptor [18–21]. Several studies have shown that its expression is decreased in multiple cancers, including squamous cell carcinoma, pancreatic cancer, gastrointestinal tract cancer and oesophageal cancer [22–25]. Through the mapping of homozygous deletions in cell lines, GNG7 was identified as a possible tumor suppressor involved in the pathogenesis of Classical Hodgkin lymphoma [26]. To investigate the mechanism, Shibata et al. transfected GNG7 into a human esophageal carcinoma cell line and found that it suppressed cell growth and tumorigenicity in BALB/c nude mice [25]. Therefore, GNG7 is a potential tumor suppressor.

Autophagy, a cellular process that degrades damaged proteins/organelles, has a debating role in cancer. Some studies show that autophagy may suppress tumorigenesis while others think autophagy promotes cancer by limiting stress responses and supporting metabolism and survival. Overall, the role of autophagy in cancer is context dependent [27, 28]. Nevertheless, autophagy is a hotspot in cancer research and a growing body of research is investigating the role and mechanism of autophagy in cancer. For this field, two autophagy markers are frequently used: LC3B and SQSTM1/p62. During autophagy activation, associated with autophagic vesicles, microtubule-associated protein light chain 3–I (LC3BI) is converted to lipidated LC3BII and displays a classical punctuate distribution. This LC3BI to LC3BII conversion, with corresponding decreases in the levels of SQSTM1/p62 (an autophagy substrate) is a classical hallmark of autophagy [29–31].

In a previous RNAi screen for cytokinesis regulators [32], we found that GNG7 RNAi led to increased binucleated cells formation, which indicates mitosis/cytokinesis failure. In the present study, we find that GNG7 is down regulated in HeLa and U2OS cancer cell lines. GNG7 overexpression can reduce HeLa cell number by increasing cell death and inhibiting cell division. GNG7 knockdown could prevent Rapamycin and EBSS-induced autophagy. We concluded that GNG7 interacted with MTOR complex and regulated autophagy through MTOR pathway.

## RESULTS

### GNG7 overexpression inhibits cell proliferation, increases cell death and blocks cells in mitosis

**Figure 1:**
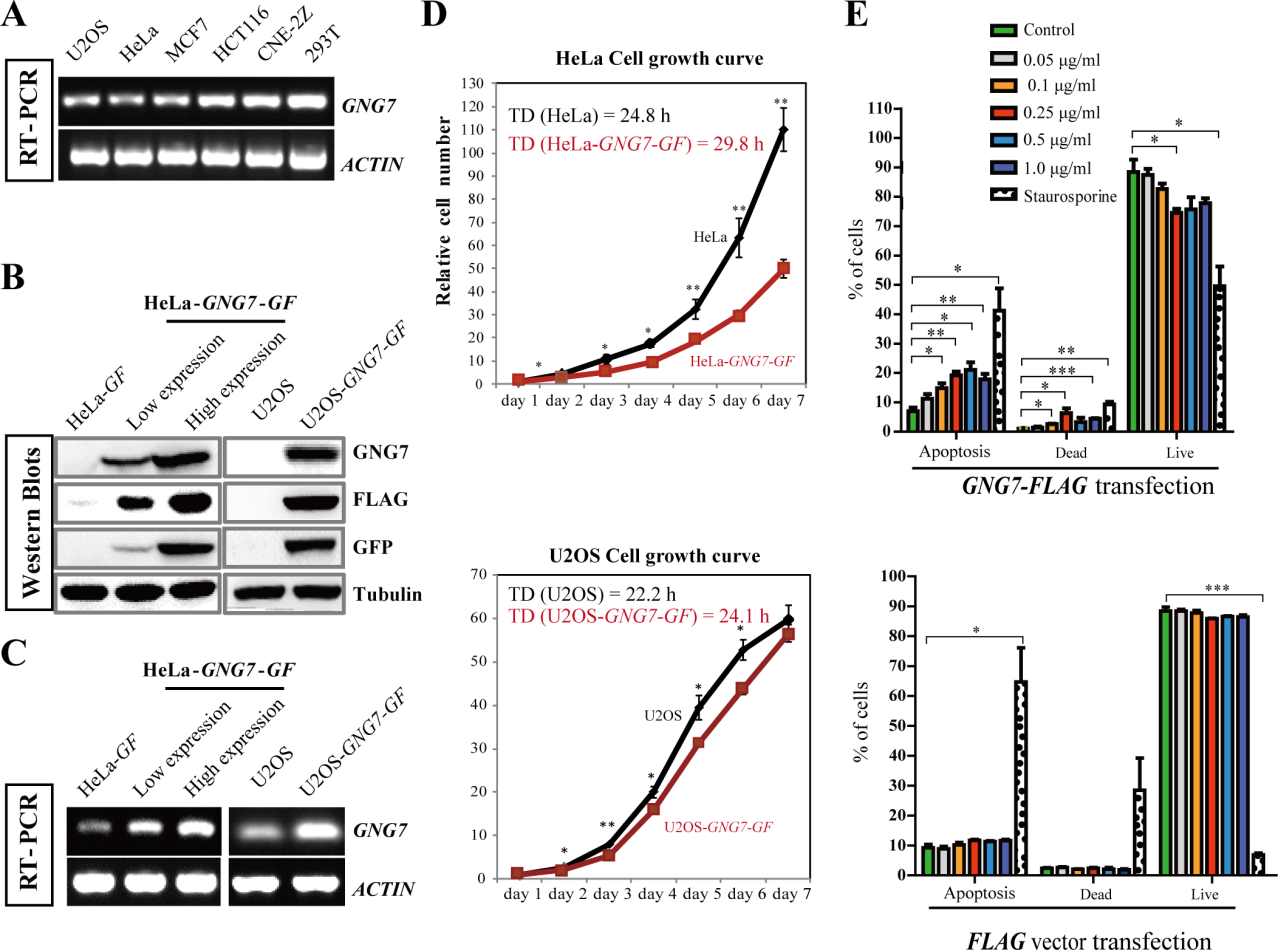
GNG7 overexpression inhibits cell proliferation and increases cell death (**A**) RT-PCR shows that the *GNG7* level is lower in U2OS, HeLa and MCF7 cells compared to HCT116, CNE-2Z and 293T cells. *ACTIN* was used as loading control. (**B**, **C**) Western blot (B) and RT-PCR (C) were used to examine the GNG7 levels in HeLa and U2OS cells that stably express GNG7-GF. Two HeLa-*GNG7-GF* stable cell lines with different expression levels were compared with HeLa-*GF* (vector control). For U2OS-*GNG7-GF* cells, U2OS was used as control. (**D**) GNG7-*GF* overexpression inhibits HeLa and U2OS cell growth. Cell growth curves and doubling times (T_D_) of HeLa, HeLa-*GNG7-GF* (high expression), U2OS and U2OS-*GNG7-GF* cell lines were compared. Data show mean ± SD. for three independent experiments. (**E**) Quantification results of Annexin/PI assays of HeLa transfected with different concentrations of *GNG7-FLAG* (top) *or FLAG vector control* (bottom). Experiments were repeated at least three times and representative results are shown. *p < 0.05, **p < 0.01, ***p < 0.001. Data show mean ± SEM. for three independent experiments.

It has been reported that GNG7 expression is down-regulated in multiple cancers [22–25], indicating that loss of GNG7 expression might contribute to carcinogenesis. We first used semi-quantitative RT-PCR to compare its expression level in a few human cancer cell lines other than the ones reported, including osteosarcoma U2OS, cervical carcinoma HeLa, breast cancer MCF7, colon cancer cell HCT116, nasopharyngeal carcinoma CNE-2Z as well as a non-cancer cell line human embryonic kidney 293T. Our results showed that the GNG7 transcriptions in the five cancer cells were lower than in 293T, especially in U2OS and HeLa cancer cell lines (Figure 1A). This and the previously published data indicate that GNG7 is likely to be generally down regulated in cancers.

To confirm the role of GNG7 in cancer inhibition, an expression vector containing the *GNG7* cDNA with *GFP* and 3 × *FLAG* tag fused at the C-terminus (*GNG7-GFP-FLAG*, or *GNG7-GF*) were transfected into U2OS and HeLa cells, the two cell lines that have significantly lower level of GNG7 compared to 293T. We obtained two HeLa cell lines that stably expressed GNG7-GF at two different levels (low expression and high expression) and one U2OS cell line that stably expressed GNG7-GF (Figure 1B and 1C). The HeLa cell line with higher GNG7 expression level was used further in this study. The growth curves of HeLa and U2OS with or without GNG7-GF expression were analyzed. The doubling times (T_D_) of HeLa and HeLa-*GNG7-GF* were 24.8 and 29.8 h, U2OS and U2OS-*GNG7-GF* were 22.2 and 24.1 h, respectively, indicating that the cell growth rates were slowed down by overexpression of GNG7 protein (*n* = 3, *p* < 0.01) (Figure 1D).

**Figure 2:**
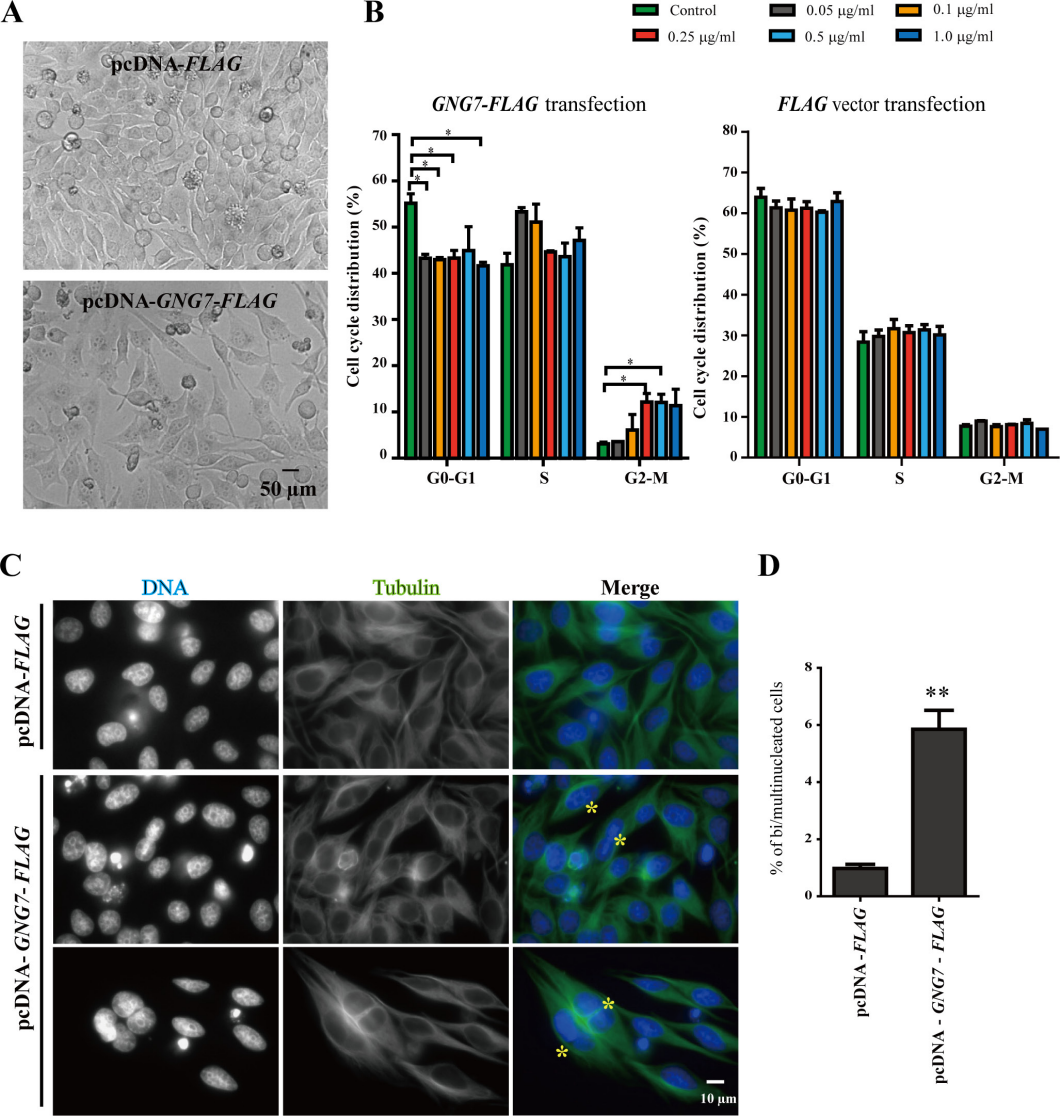
GNG7 overexpression arrests cells in M phase HeLa cells were transfected with *FLAG* vector control or *GNG7-FLAG* for 48 hours before they were directly imaged by bright field microscopy (**A**) or harvested for cell cycle analysis using flow cytometry (**B**), or immunofluroscence using anti-Tubulin antibody (green) and DAPI (blue) (**C**). 0.5 μg/ml of *FLAG* or *GNG7-FLAG* plasmids were used in (A) and (C). The yellow “*” indicates bi/multinucleated cells. (**D**) Quantification of bi/multinucleated cell percentage in (C) from three independent experiments. Experiments were repeated at least three times and representative results are shown. *p < 0.05, **p < 0.01. Data show mean ± SEM, *n* = 3.

The reduced cancer cell number can be either due to increased cell death or reduced cell division/proliferation. We first examined whether GNG7 affected apoptosis. Here we made another construct for transient transfection, *GNG7-FLAG,* instead of *GNG7-GFP-FLAG (GNG7-GF)* to avoid the possibility of the possible interference of the GFP tag. Annexin and PI dual labeling and flow cytometry were used to examine the effects of *GNG7-FLAG* transient transfection in HeLa cells. Staurosporine was used as a positive control. Our results showed that after transfection with 0, 0.05, 0.1, 0.25, 0.5, or 1.0 μg/ml *GNG7-FLAG* plasmids, but not the *FLAG* vector control, for 48 hours, the apoptotic and dead cells increased in a dose-dependent manner (Figure 1E and Figures S1, S2), which indicates that GNG7 induces cell death to inhibit cancer. However, it should be noted that the cell numbers of HeLa cells transfected with *GNG7-FLAG* plasmid for 48 hours were at least reduced by half compared to vector control (Figure 2A), while the proportion of apoptotic cells was no more than 20%. This indicates that induced cell death is not the only reason for the cell number reduction. We then collected HeLa cells transfected with 0, 0.05, 0.1, 0.25, 0.5 and 1.0 μg/ml *GNG7-FLAG* plasmids or vector control for flow cytometry assays. We found that G2-M population was increased in a dose-dependent manner after *GNG7-FLAG* overexpression, and the G0–G1 cells were decreased simultaneously (Figure 2B and Figure S3). In contrast, the vector control overexpression did not affect cell cycle (Figure 2B). In addition, even the highest concentration, 1.5 μg/ml of *GNG7-FLAG* plasmid did not lead to cell senescence (Figure S4). This indicates that GNG7 expression induces cell cycle arrest to decrease cell number. Therefore GNG7 induces both cell death and cell cycle arrest to reduce cell number.

The increased G2-M phase cells could be resulted from arrested at the stage of G2 or at M phase. To differentiate these two possibilities, we used immunofluroscence to examine the cells transfected with *GNG7-FLAG*. We found that GNG7 overexpression induced binucleated cells (Figure 2C and 2D), which were usually caused by abnormal mitosis and/or cytokinesis. These results indicate that GNG7 blocks cells in mitosis or cytokinesis to inhibit cell growth.

### GNG7 knockdown induces bi/multinucleated cells and reduces cell number

**Figure 3:**
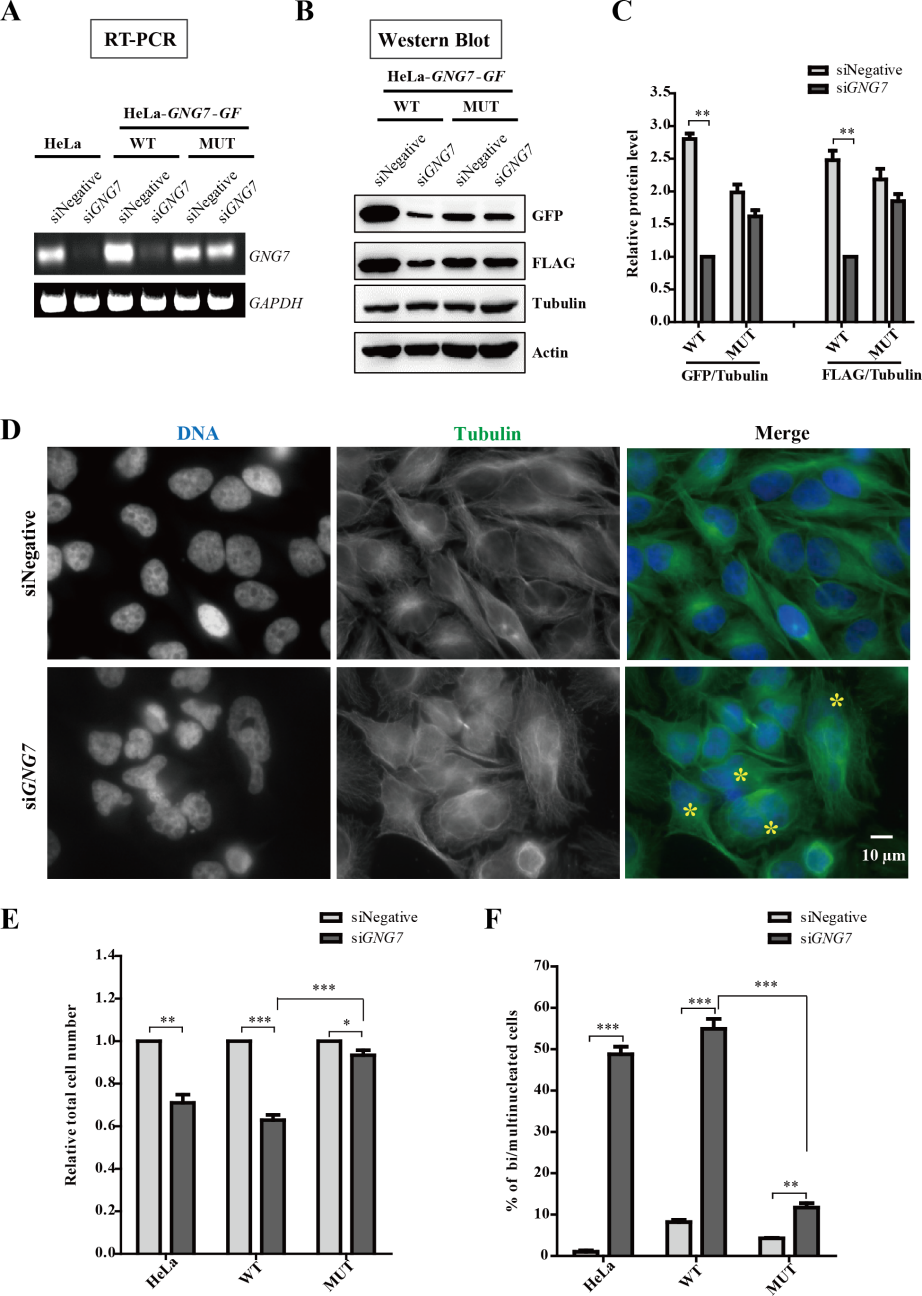
GNG7 RNAi induces bi/multinucleated cells and reduces cell number (**A–C**) GNG7 RNAi specifically knockdown GNG7. HeLa, HeLa-*GNG7-GF*-WT and HeLa-*GNG7-GF*-MUT (RNAi resistant mutant) cells were transfected with control or *GNG7* siRNAs for 72 hours before they were lysed analyzed for RT-PCR (B) and Western blots (C, D). (**D**) GNG7 RNAi induces bi/multinucleated cells. HeLa cells were transfected with siRNAs for control or *GNG7* for 72 hours before they were fixed and stained for microtubules and DNA with anti-tubulin antibody (green), and DAPI (blue). “*” indicates bi/multinucleated cells. (**E, F**) Quantification results show that GNG7 RNAi reduces cell number (E) and increases bi/multinucleated cell numbers (F). Experiments were repeated three times and representative results are shown. *p < 0.05, **p < 0.01, ***p < 0.001. Data show mean ± SEM. for three independent experiments.

To unveil the mechanism by which GNG7 affects cell division, we performed RNAi experiment to analyze the effect of GNG7 knockdown on cell division. In addition, to validate the RNA knockdown specificity, we constructed an RNAi resistant mutant expression vector for si*GNG7* (si*GNG7*-1, si#1,) and a stable cell line HeLa-*GNG7-GF* MUT (MUT). As expected, GNG7 RNAi specifically reduced *GNG7* RNA and protein in both HeLa and HeLa-*GNG7-GF* WT cells, but not in HeLa-*GNG7-GF* MUT (Figure 3A and 3B; Figure S5). GNG7 expression level was reduced by around 65% and 60% as measured by GFP and FLAG antibodies individually (*p* < 0.01) in HeLa-*GNG7-GF* WT cells, while the RNAi resistant mutant HeLa-*GNG7-GF* MUT cells still had 83% of GNG7 protein remaining compared to siNegative control (Figure 3C).

We found that GNG7 knockdown significantly increased the number of bi/multinucleated cells and reduced cell number (Figure 3D and Figure S5). Quantification results showed that in HeLa and HeLa-*GNG7-GF* WT cells, the total cell numbers were decreased to 70%, 60% by si*GNG7* treatment (Figure 3E). At the same time, the percentage of bi/multinucleated cells increased from 1% to 48% in HeLa cells and from 8% to 54% in WT cells after GNG7 RNAi (Figure 3F). The RNAi resistant mutant partially rescued GNG7 RNAi phenotype, which was because the transfected protein level was too low in some cells. In addition, its resistant effects were statistically significant compared to HeLa-*GNG7-GF* WT cells (*n* = 3, *p* < 0.001). To further analyze the specificity of GNG7 in cell division, we treated HeLa cells with Pertussis toxin (PTX) for 72 hours, which inactivated all members of the Gα_i_ family of G proteins, and found that even at high concentrations of PTX, 0.5 and 1.0 μg/ml, there were only a slight increase of binucleated cells (Figure S6). These experiments suggest that GNG7 plays an important role in cell division.

It seems contradictory that both GNG7 overexpression and knockdown can both lead to decreased cell numbers and binucleated cells. Actually, this is not hard to understand because we found in the next session that GNG7 affects actin polymers, increasing or decreasing of which both could affect cell division. This is similar to the microtubule polymers because stabilizing (such as Taxol) or destabilizing (such as Nocodazole) can both cause mitotic arrest and cell death.

### GNG7 expression is cell cycle dependent and it affects cell division by disrupting actin cytoskeleton

**Figure 4:**
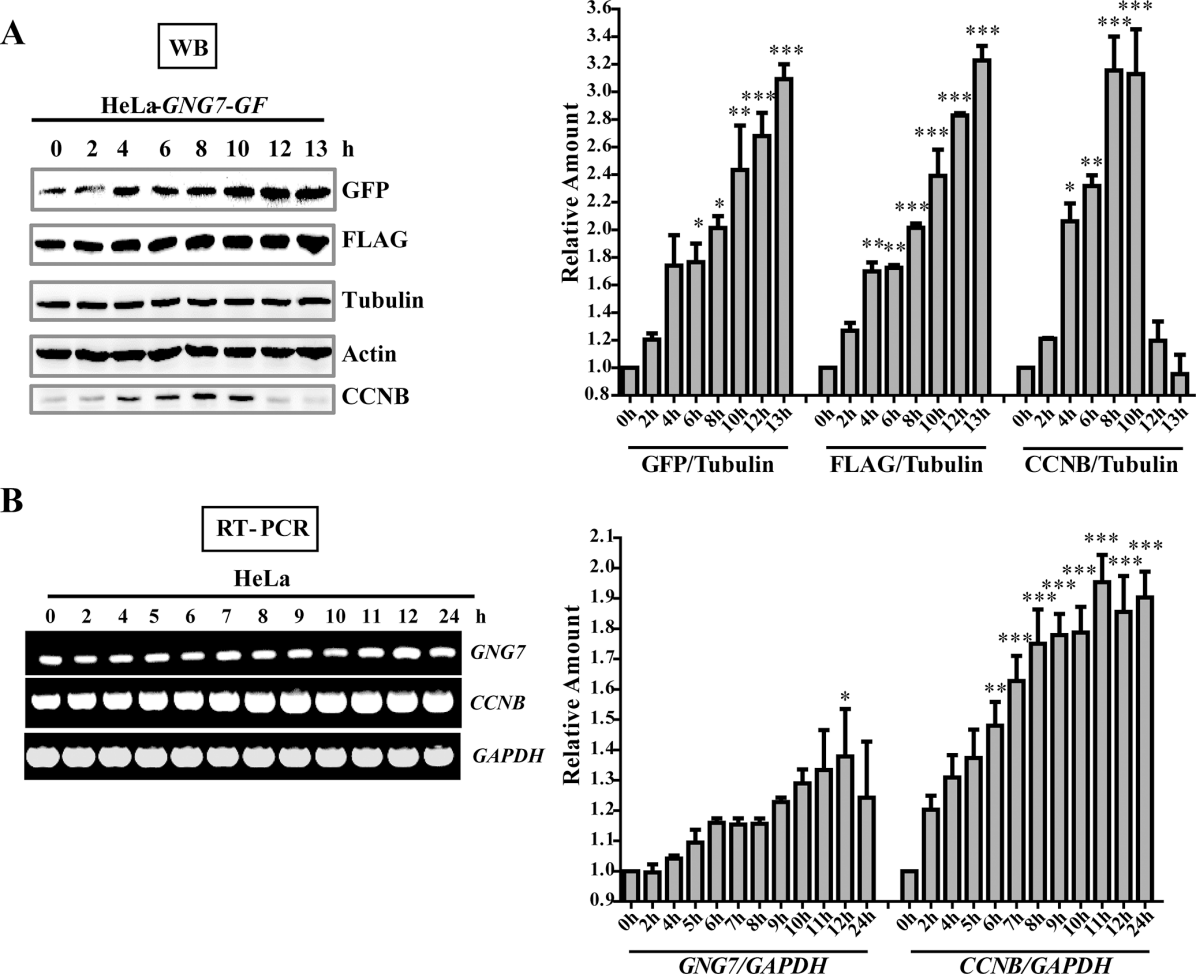
GNG7 level is increased in mitosis (**A**) Western blot analysis shows that GNG7 is increased in mitosis. HeLa*-GNG7-GF* cells were synchronized by double thymidine block before they were released for 0, 2, 4, 6, 8, 10, 12 and 13 hours. Cells were lysed for western blotting assay with GFP and FLAG antibodies. Tubulin and Actin were used as loading controls. (**B**) RT-PCR analysis shows that *GNG7* is increased in mitosis. Double thymidine synchronized HeLa cells were released for 0, 2, 4, 5, 6, 7, 8, 9, 10, 11, 12 and 24 hours before they were harvested for RT-PCR. *CCNB* was used as a mitotic marker. Experiments were repeated three times and representative results are shown. Quantifications on the right were done using One-way ANOVA with post-Dunett analysis by GraphPad Prism5. *p < 0.05, **p < 0.01, ***p < 0.001.

To further assess the role of GNG7 in cell division, we first analyzed its expression level in different cell cycle stages using Western blotting and semi-quantitative RT-PCR in synchronized cells. The GNG7 expression in HeLa cells is too low to be detected with the GNG7 antibody, so we used HeLa-*GNG7-GF* cell line for further analysis. CCNB (Cyclin B) is a regulatory protein involved in early events of mitosis and was used as a frequently used mitotic marker. Both our Western blot and RT-PCR results show that GNG7 is up-regulated at the same time with CCNB during the cell cycle (Figure 4). And with the progression of mitosis, the protein level of GNG7 continued to increase. Semiquantitative RT-PCR also showed *GNG7* transcription level was increased until 12 hours from thymidine release (*p* < 0.05, *n* = 3) (Figure 4B). These confirmed that the effect of GNG7 on regulating cell division might be relevant to its function in mitosis.

**Figure 5:**
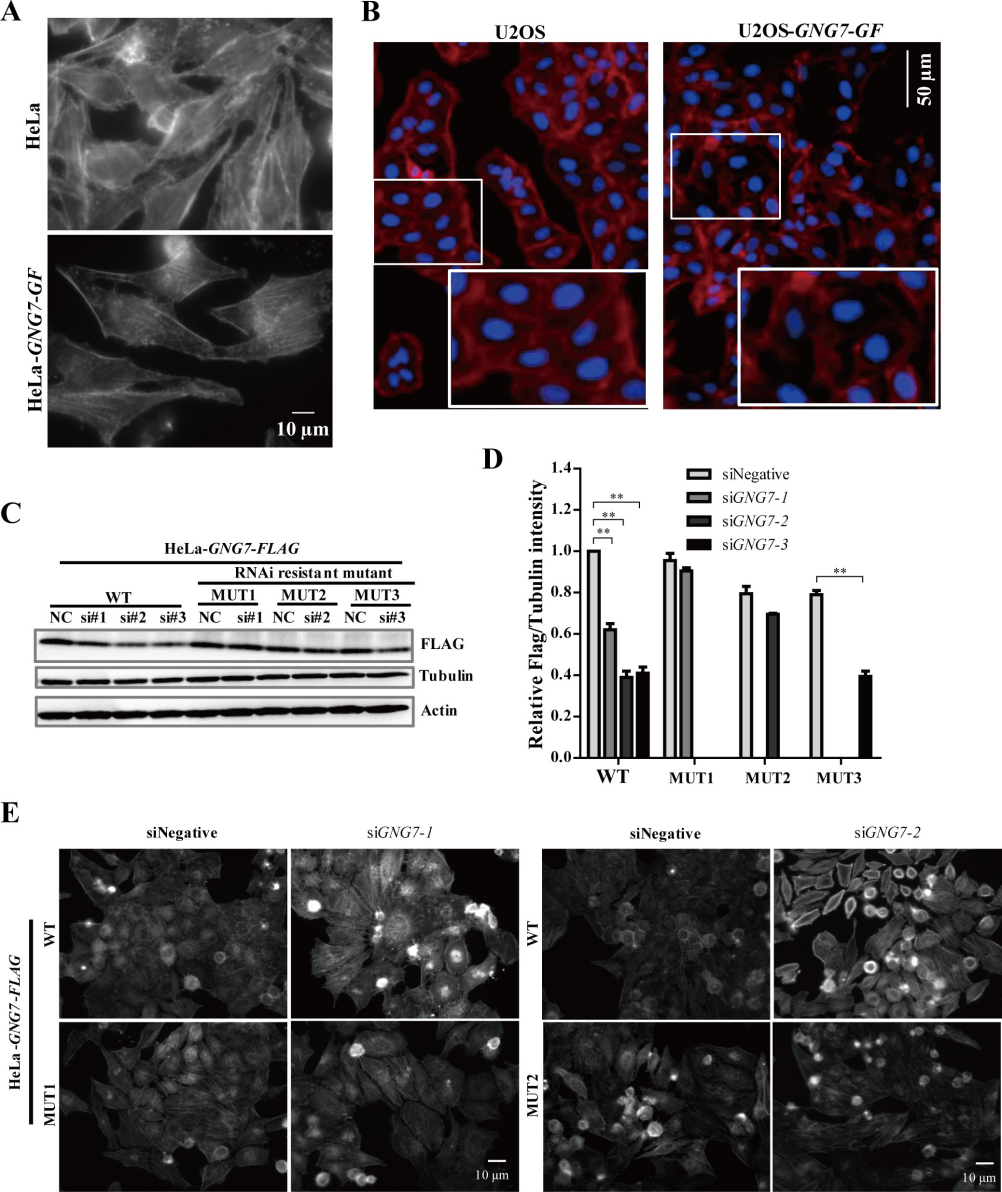
GNG7 overexpression reduces actin polymer and GNG7 RNAi increases actin polymer in cells (**A, B**) Actin polymer staining shows that GNG7 overexpression reduces actin polymer in cells. (A) HeLa-*GNG7-GF* and (B) U2OS-*GNG7-GF* stable cell lines were fixed and stained with fluorescence-labeled phalloidin. (**C**, **D**) GNG7 RNAi specificity. HeLa, HeLa-*GNG7-FLAG* WT and HeLa-*GNG7-FLAG* RNAi resistant mutant (MUT1, 2, 3) stable cell lines were transfected with control or different *GNG7* siRNAs for 72 hours before they were analyzed by Western blots. MUT1, 2, 3 were designed to be resistant for siRNA *GNG7*-1, *7*-2, or *7*-3, respectively. (**E**) GNG7 RNAi-induced actin phenotype can be rescued by RNAi resistant mutant. HeLa-*GNG7-FLAG* WT and RNAi resistant MUT1 or MUT2 stable cell lines were transfected with siRNAs for control, *GNG7*-1 or *GNG7*-2 for 72 hours before they were fixed and stained with fluorescence-labeled phalloidin. Results are not shown for si*GNG7-1* and MUT1. Experiments were repeated three times and representative results are shown. **p < 0.01. Data show mean ± SEM. for three independent experiments.

Reorganization of the actin cytoskeleton during mitosis was reported crucial for regulating cell division [33–35]. To examine whether GNG7 affects actin, we examined actin cytoskeleton by immunofluorescence in GNG7 overexpression and knockdown cells using fluorescently labeled phalloidin, which specifically binds actin polymers (F-actin). GNG7-GF overexpression decreased the actin fibers both in HeLa (Figure 5A) and U2OS cells (Figure 5B), which indicated that GNG7 affected actin cytoskeleton by decreasing actin polymer. To further confirm this hypothesis and examine the specificity, three siRNAs for *GNG7* were examined in HeLa, HeLa-*GNG7-FLAG* WT and three RNAi resistant mutant cell lines (MUT1, MUT2, and MUT3, resistant to siRNA#1, 2, 3 respectively) for 72 hours. All three siRNAs can reduce GNG7 expression level by about 50% (*p* < 0.01) in HeLa-*GNG7-FLAG* WT cells (Figure 5C and 5D). MUT1 and MUT2 cell lines could efficiently rescue siRNA#1 and 2, while MUT3 only had partial rescue effect (Figure 5C and 5D). Therefore we chose the first two siRNAs for further study. We found that both siRNAs caused increased F-actin in HeLa and HeLa-*GNG7-FLAG* WT cells, which could be rescued by the two RNAi resistant mutants (Figure 5E). These results confirmed that GNG7 RNAi could increase F-actin in cells, which is likely a major reason for its involvement in cell division.

### GNG7 induces autophagy

**Figure 6:**
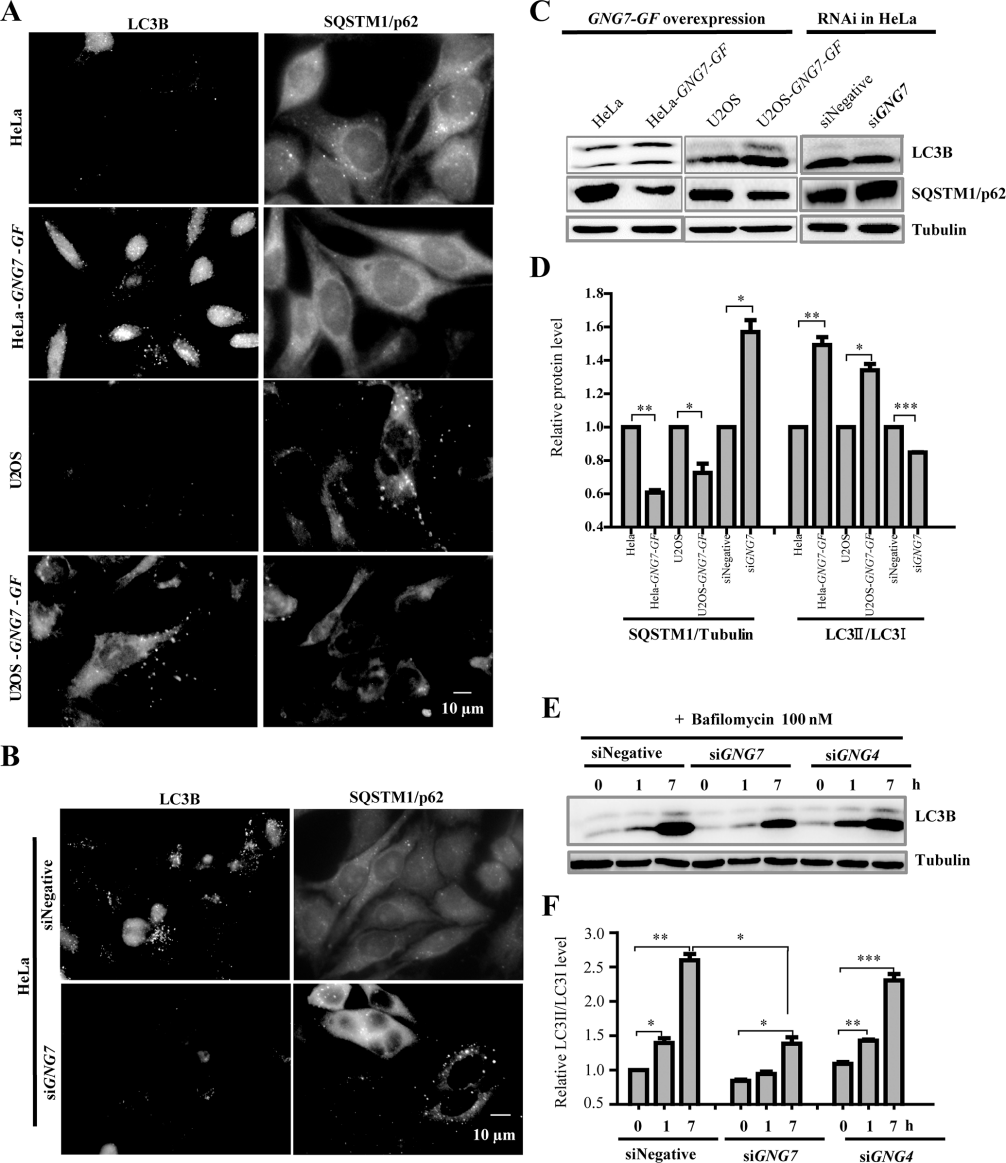
GNG7 induces autophagy in cells (**A**, **B**) Immunofluorescence of LC3B and p62 in (A) HeLa, HeLa-*GNG7-GF*, U2OS and U2OS-*GNG7-GF* cells or (B) HeLa cells treated with control or *GNG7* RNAi. (**C**) Western blot analysis for LC3B and p62 level in HeLa, HeLa-*GNG7-GF*, U2OS and U2OS-*GNG7-GF* cells as well as HeLa cells treated with control or *GNG7* siRNA. (**D**) Quantification of (C). (**E**, **F**) Western blot analysis shows that GNG7 RNAi, but not GNG4 RNAi, decreases autophagy level in cells. HeLa cells were transfected with control, *GNG7* or *GNG4* siRNAs for 72 hours before they were treated with DMSO or 100 nm Bafilomycin (BAF) for 1 or 7 hours and harvested for Western blots. (F) Quantification of (E). Experiments were repeated at least three times and representative results are shown. *p < 0.05, **p < 0.01, ***p < 0.001. Data show mean ± SEM, *n* = 3.

An unexpected observation was that GNG7 affected autophagy in cells when our lab was conducting some other autophagy researches and used GNG7 siRNA as a related control. As shown in Figure 6A, GNG7 overexpression increased LC3 puncta and decreased SQSTM1/p62 in both HeLa and U2OS, indicating increased autophagy level. Conversely, GNG7 RNAi led to increased SQSTM1/p62 puncta and decreased LC3B puncta (Figure 6B), indicating decreased autophagy level. Western blot analysis also showed that GNG7 overexpression caused decreased SQSTM1/p62 (about 40% in HeLa, *p* < 0.05; 30% in U2OS, *p* < 0.01) and increased LC3BII/I (about 50% in HeLa, *p* < 0.05; 30% in U2OS, *p* < 0.01) (Figure 6C and 6D). Consistently, GNG7 RNAi caused increased SQSTM1/p62 (150%, *p* < 0.05) and decreased LC3BII/I (84%, *p* < 0.01). These results suggest that GNG7 induces autophagy.

To confirm the inducer role of GNG7 in autophagy, we used a well established autophagy assay by comparing the LC3BII/I ratio change in the presence or absence of Bafilomycin (BAF), which blocked autophagy at a late stage by inhibiting the fusion between autophagosomes and lysosomes. BAF treatment alone increased LC3BII/I as expected because it could prevent autophagosomes degradation and accumulate autophagosome numbers. We found that GNG7 knockdown efficiently reduced BAF-induced LC3BII/I increase from 2.6 fold to 1.4 fold (*p* < 0.05) (Figure 6E and Figure 6F), which suggests that GNG7 knockdown decreases the autophagy flux in cells. We further analyzed the specificity of GNG7 in autophagy by checking another heterotrimeric G protein, G protein γ subunit 4 (GNG4). Although GNG4 RNAi can efficiently knockdown GNG4 (Figure S7A), it does not affect autophagy (Figure 6E and Figure 6F) or cell division (Figure S7B). These demonstrate that GNG7 is an autophagy inducer and not all γ subunits of G protein are involved in autophagy.

### GNG7 inhibits MTOR pathway and induces autophagy

**Figure 7:**
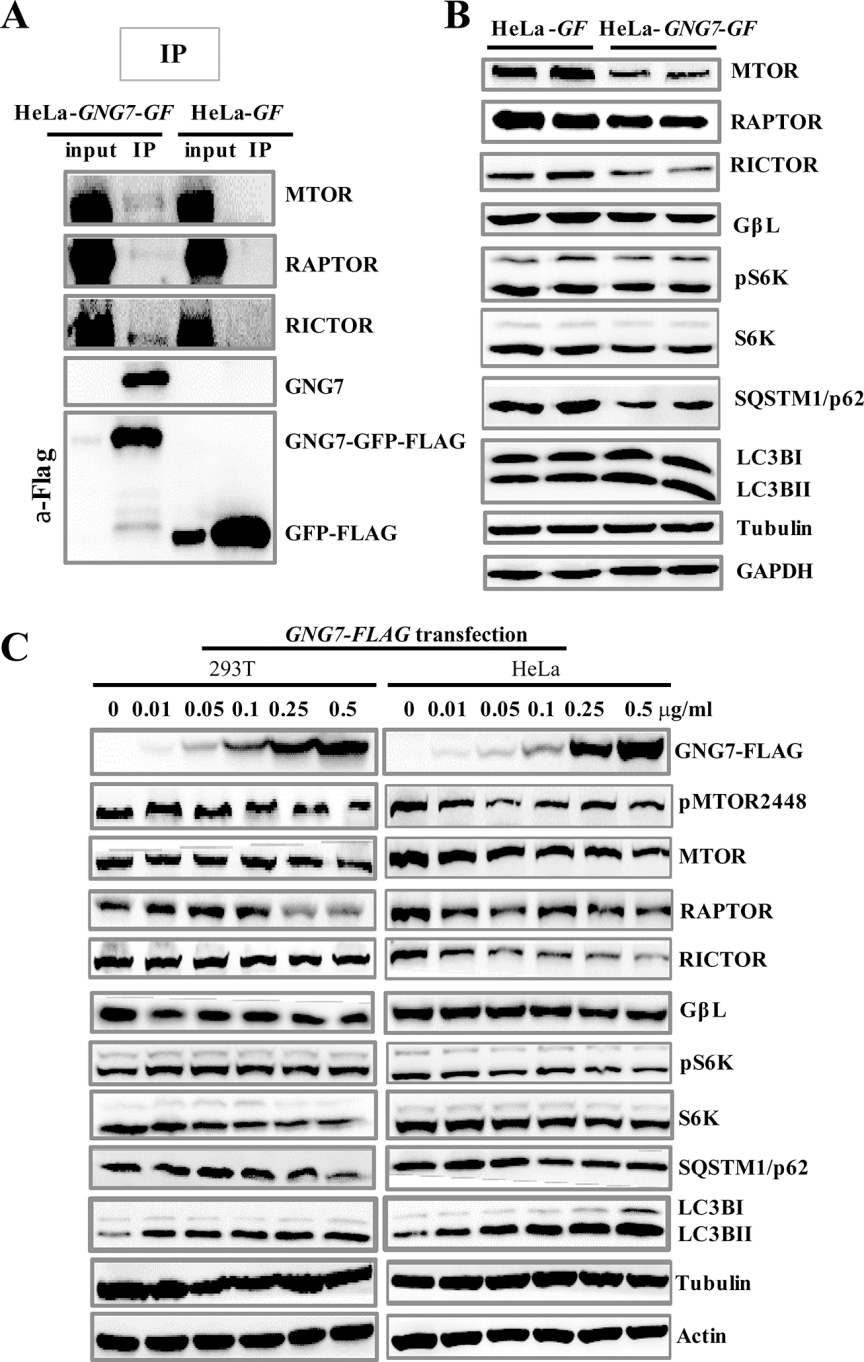
GNG7 inhibits MTOR pathway and induces autophagy (**A**) Immunoprecipitation shows that GNG7 interacts with MTOR complex. HeLa-*GF* and HeLa-*GNG7-GF* stable cell lines were harvested and immunoprecipitated with anti-FLAG antibody. Anti-MTOR, RAPTOR, RICTOR, GNG7 and FLAG antibodies were used in Western blots. The input/IP loading ratio was 1/20. (**B**) Western blot analysis to compare protein levels in HeLa-*GF* and HeLa-*GNG7-GF* cells. (**C**) HeLa and 293T cells were transfected with 0, 0.01, 0.05, 0.1, 0.25, and 0.5 μg/ml of *GNG7-FLAG* plasmid for 48 hours before they were harvested for Western blots. Experiments were repeated two times and representative results are shown.

MTOR is a central regulator of autophagy and AKT/MTOR pathway signals from growth factors, nutrients and stresses to control cell metabolism, survival and proliferation [36, 37]. To unravel the possible link between GNG7-induced autophagy with MTOR, we first used co-immunoprecipitation experiments to check whether they interact. Using HeLa-*GF* (vector control) and HeLa-*GNG7-GF* cell lines, we found that GNG7 indeed interacted with MTOR complexes, including MTOR, RAPTOR, and RICTOR (Figure 7A), although the interaction is fairly weak.

To rule out the possible false positive of the immunoprecipitation experiment, we next determined that if the MTOR pathways were affected by GNG7 in cells. Western blot results showed MTOR pathway signaling was significantly inhibited in GNG7 overexpression cells (Figure 7B). Decreased phosphorylations of MTOR and p70S6K (S6K), the key downstream effector of MTOR, were detected in HeLa-*GNG7-GF* cells, along with the reduced levels of MTOR, RAPTOR, RICTOR and S6K proteins (Figure 7B). To confirm the impact of GNG7 on MTOR pathways, we did transient transfection of an expression vector of *GNG7* into HeLa and 293T cells respectively. Western blot results showed *GNG7-FLAG* transfection resulted in a dose-dependent decrease in a few MTOR pathway proteins, as well as SQSTM1/p62 decrease and LC3B increase (Figure 7C). These results indicate that GNG7 inhibits MTOR pathway to increase autophagy.

**Figure 8:**
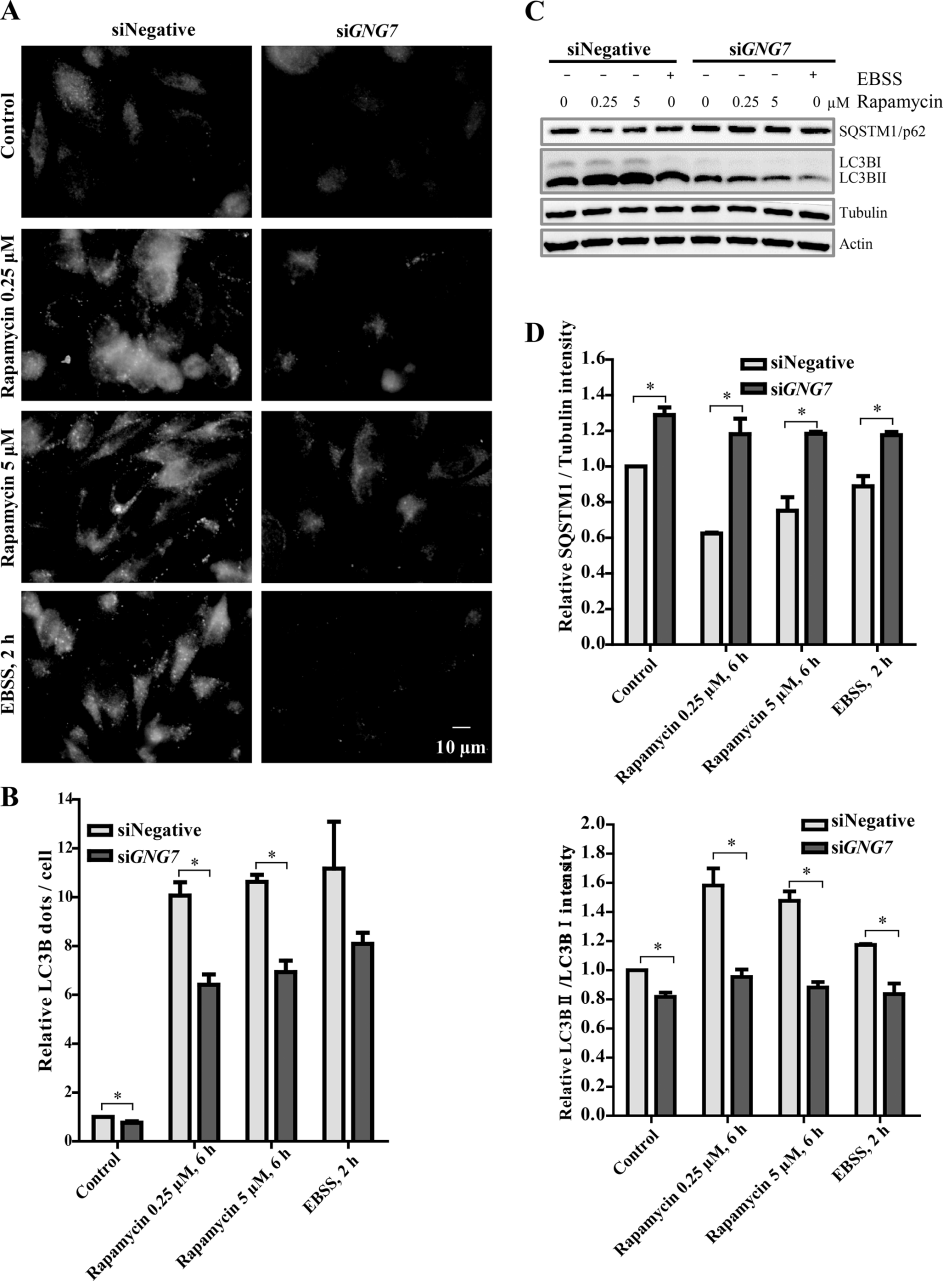
GNG7 knockdown reduces Rapamycin and EBSS induced autophagy HeLa cells were transfected with siRNAs for control or *GNG7* and cultured for 72 hours. Before harvesting, cells were treated with DMSO, 0.25 μM or 5 μM Rapamycin for 6 hours, or EBSS for 2 hours. (**A**) Immunofluorescence shows that GNG7 knockdown reduces Rapamycin and EBSS-induced LC3B dots. (**B**) Quantification of LC3B dots/cell in (A). (**C**) Western blot analysis shows that GNG7 knockdown reduces Rapamycin and EBSS-induced autophagy. SQSTM1/p62, LC3B and Tubulin antibodies were used. (**D**) Quantification of p62/tubulin and LC3BII/LC3BI in (C). Data represents three independent experiments and are presented as mean ± SEM. *p < 0.05.

Further analysis revealed that GNG7 knockdown reduced Rapamycin, a MTOR inhibitor, and starvation-induced autophagy (Figure 8). HeLa cells transfected with siRNAs for control and *GNG7* were treated with DMSO, 0.25 μM or 5 μM Rapamycin for 6 hours or EBSS medium for 2 hours before harvested. Immunofluorescence showed that the LC3B puncta per cell was increased by Rapamycin or EBSS because they both work as autophagy inducers. However, GNG7 knockdown reduced Rapamycin-induced LC3 puncta (Figure 8A and 8B). Western blot results showed that Rapamycin and EBSS-induced SQSTM1/p62 clearance and LC3BII/I increase were effectively reduced by GNG7 RNAi (Figure 8C and 8D). For example, GNG7 knockdown reduced 5 μM Rapamycin-induced LC3BII/I from 150% to 108% (*p* < 0.05).

## DISCUSSION

GNG7 is the first reported Gγ protein that functions in autophagy. Gα proteins were shown to participate in autophagy and most studies focused on Gα_i3_ [38–43]. Recently, Yuan's group used siRNAs to examine multiple Gα proteins, including Gα_s_, Gα_q11_ and Gα_12/13_, and found that their knockdown could induce autophagy. Although it is evident that different groups of G protein may have different roles, no evidence so far shows that Gβ or γ proteins are involved in autophagy. At the same time, studies of the Gα proteins show that they are autophagy inhibitors. For example Gα_s_, Gα_q11_ and Gα_12/13_ knockdown can induce autophagy. However, our study shows that GNG7 is an autophagy inducer, which is different than Gα proteins. This is not too surprising because Gβγ is thought as a negative regulator of Gα signaling, and can decrease the signal-to-noise ratio by preventing spontaneous Gα activation [44]. In this view, the roles of Gβγ in autophagy may be opposite to Gα.

Different subunits within the same group may have distinct functions. For example, our study show that GNG7 overexpression in HeLa and U2OS cells reduces actin stress fibers and its knockdown increases actin polymer, which indicates that GNG7 functions in actin cytoskeleton regulation. However, GNG4, a different Gγ does not have this function. In addition, it has been reported that Gβ_1_γ_12_ has a much more striking effects on actin stress fibers than Gβ_1_γ_7_ induced stress fiber disruption [45, 46]. Although they investigated the Gβ_1_γ_7_ and Gβ_1_γ_12_ as dimers and GNG7 was known to coordinate with Gβ_2_ but not Gβ_1_ in cells [18, 20], their differential phenotypes indicate that Gγ_12_ has a more important role than Gγ_7_ in actin regulation. This is very interesting because Gγ_7_ and Gγ_12_ are the most similar subunits in the Gγ group [47]. Further understanding of how a G protein subunit differs from each other at a molecular level is crucial to G protein related cellular events.

Our data show that the potent cell growth inhibition effect of GNG7 overexpression is likely combined effects of autophagic cell death and cell division inhibition. Although our results demonstrate that GNG7 is an important player in both autophagy and cell division, whether it regulates these two cellular processes by the same mechanism is still not clear. Our experiments show that GNG7 interacts with and inhibits MTOR, which is likely the major reason that GNG7 induces autophagy. It has been reported that Gβγ heterodimers containing different Gβ (Gβ_1–5_) subunits with γ_2_ exhibiting interaction with MTOR except Gβ_4_ [48], which not only revealed the link between Gβγ and MTOR but also showed that different subunits had differential functions. Meanwhile, GNG7-induced F-actin decrease should be the reason for binucleated cell formation in GNG7 overexpression or knockdown cells, since either increase or decrease F-actin can affect cell division. It has been shown that in *Dictyostelium discoideum,* ElmoE associated with the Dicty Gβγ subunit and interacted with Dock-like proteins to promote actin polymerization in cell migration [49], whether GNG7 uses the same mechanism in mammalian cells to regulate actin cytoskeleton and cell division will need further investigations. In addition, It should be mentioned that although GNG7 protein level increases together with CCNB during G2 and early mitosis, it does not decrease in later stages of mitosis as CCNB protein does. Its protein levels in later stages of mitosis/cytokinesis are actually higher than earlier stages of mitosis. The exact role of GNG7 at different stages of cell division also needs to be further investigated.

In conclusion, our study shows that GNG7 affects actin cytoskeleton and inhibits MTOR pathway in cells, which lead to inhibited cell division, increased cell death and autophagy. These effects all contribute to the anti-tumor effect of GNG7. Further analysis is needed to investigate the tumor repressing role of GNG7 in a broader application and the mechanism by which GNG7 interacts and regulates MTOR signaling pathway.

## MATERIALS AND METHODS

### Cell culture

HeLa, U2OS, MCF7, HCT116, and 293T cells were grown in monolayers in DMEM without L-glutamine (Coring Life Sciences) supplemented with 10% (v/v) fetal bovine serum (FBS; Pufei Biotechnology, Shanghai, China), 1% (v/v) penicillin/streptomycin (P/S; Hyclone), 1% glutamax (Gibco, Life Technologies), 5% CO_2_, at 37°C. HeLa-*GNG7-GFP-FLAG* wild type (HeLa-*GNG7-GF* WT), HeLa-*GNG7-GFP-FLAG* RNAi resistant mutant (HeLa-*GNG7-GF* MUT), HeLa-*GNG7-FLAG* wild type (HeLa-*GNG7-FLAG* WT), HeLa-*GNG7-FLAG* RNAi resistant mutant (MUT1, MUT2 and MUT3), and U2OS-*GNG7-GFP-FLAG* wild type (U2OS-*GNG7-GF*) cells were maintained similar to HeLa cells, with addition of 1 μg/ml puromycin (Invitrogen) in DMEM. CNE-2Z cell line was cultured in RPMI-1640 supplemented with 10% (v/v) FBS and 1% (v/v) P/S.

### Wild type and RNAi-resistant *GNG7-GFP-FLAG* and *GNG7-FLAG* stable cell lines

The cDNA encoding human GNG7 was cloned into pMSCV-puro vector with a *GFP* tage and a 3 ×*FLAG* tag fused at the C-terminal to form *GNG7-GFP-FLAG* (*GNG7-GF*) plasmid. Plasmid *GNG7-FLAG* was constructed by ligating the *GNG7* cDNA into pMSCV-puro vector with 3×*FLAG* tag at C-terminal. Retroviruses of *GFP-FLAG (GF), GNG7-GF*, and *GNG7-FLAG* were packaged by transfecting the plasmids with two helper plasmids into 293T cell lines using TransIT (Mirus). Then the supernatants were harvested after 48 hours incubation. Stable cell lines were established by infection of HeLa and U2OS cells with retroviruses and screened by puromycin in a final concentration of 1 μg/ml.

RNAi-resistant *GNG7-GF* MUT and *GNG7-FLAG* MUT1 constructs were obtained by silent mutagenesis at the 105th-123rd region (si*GNG7*/si*GNG7*-1/si#1 targeted region) CATGAGCTACTGTGAGCAA→TATGTCATATTGCGAACAG with 42% mismatch. Silent mutagenesis regions of two other RNAi-resistant *GNG7-FLAG* constructs were GAGCGCATCAAGGTCTCCAAA (the 72nd-90th nucleotides, si*GNG7*-2/si#2 targeted region)→GAAAGGATTAAAGTGAGTAAG for MUT2 and GTCTGACCTCATGAGCTAC (the 96th-114th nucleotides, si*GNG7*-3/si#3 targeted region)→TAGCG ATCTGATGTCATAT for MUT3, both with 53% mismatch. Stable cell lines were made in HeLa as described above.

### Wild type and RNAi-resistant *GNG4-GFP-FLAG* stable cell lines

The cDNA encoding human GNG4 was cloned into the same pMSCV-puro vector as GNG7 to form *GNG4-GFP-FLAG* (*GNG4-GF*) plasmids. RNAi-resistant *GNG4-GF* MUT was obtained by silent mutagenesis at the 73th-93th region ATGGAAGCCTGTATGGACAGG to ATGGAGGCATGCATGGATCGT with 29% mismatch. *GNG4-GF* WT and MUT stable cell lines were made in HeLa as described above.

### Transient transfection

In order to improve the transient transfection efficiency, *GNG7* cDNA was cloned into pcDNA3.1 vector with *FLAG* tag fused at the C-terminal to form *GNG7-FLAG* plasmid. HeLa or 293T cells were plated at 1 × 10^6^ cells/cm^2^ density. After 12 hours cultivation, cells were transfected with 0, 0.01, 0.05, 0.1, 0.25, 0.5 and 1.0 μg/ml *GNG7–FLAG* or *FLAG* plasmids using lipofectamine 2000 (Invitrogen), and cultured for further 48 hours. Then the supernatant and cells were collected for immunofluorescence, western blotting, flow cytometry for cell cycle and Annexin V/PI apoptosis detection assay.

### RNAi

HeLa, HeLa-*GNG7-GF*, HeLa-*GNG7-FLAG* or RNAi-resistant mutant cells were plated in 12-well or 24-well plates. SiRNAs for *GNG7*, *GNG4* or negative control were transiently transfected using Hiperfect (Qiagen) following the manufacture's protocol at 40 nM. After 72 hours incubation, cells were either lysed for Western Blotting or RT-PCR, or fixed to perform immunofluorescence experiments. U2OS and U2OS-*GNG7-GF* cells were used following the same protocol to verify the results. To further confirm the cytoskeleton results, si*GNG7*-2 and si*GNG7*-3 were employed to repeat the experiments in HeLa-*GNG7-FLAG* or RNAi-resistant mutant stable cell lines.

The sequences of siRNA oligos (Genepharm) used were as below: 5′–3′.

SiNegative or NC: UUCUCCGAACGUGUCACG UTT.

Si*GNG7*, si*GNG7*-1 or si#1: CAUGAGCUACUGU GAGCAA.

Si*GNG7*-2 or si#2: GAGCGCAUCAAGGUCU CCAAA.

Si*GNG7*-3 or si#3: GUCUGACCUCAUGAGCUAC.

Si*GNG4*: ATGGAAGCCTGTATGGACAGG.

### RT-PCR

Cells were washed once with phosphate buffered saline (PBS). Total RNA was extracted following a modified guanidinium thiocynate method using Trizol (Invitrogen). cDNA was synthesized using PrimeScript^®^ RT reagent Kit (Takara). *GNG7* and *CCNB* genes were amplified from prepared cDNAs using fast pfu DNA polymerase (TransGen Biotech) with the following primers listed in Table 1. DNA levels were normalized to the matching densitometric value of the internal control *GAPDH* or *ACTIN* by ImageJ software.

**Table 1:** RT-PCR primers (5′-3′)

*GNG7*F	CGTCTGACCTCATGAGCTACTGTGA
*GNG7*R	CAAGGTTTCTTGTCCTTAAAGGGGTTC
*CCNB*F	GACTGGCTAGTACAGGTTCAAATGAAAT
*CCNB*R	GTTCTTGACAGTCATGTGCTTTGTAAGT
*GAPDH*F	GTCACCAGGGCTGCTTTTAACTCT
*GAPDH*R	GGGTCTCTCTCTTCCTCTTGTGCT
*ACTIN*F	AGATCATGTTTGAGACCTTCAACACC
*ACTIN*R	GCAATGATCTTGATCTTCATTGTGC

### Immunofluorescence

HeLa, HeLa-*GNG7-GF*, HeLa-*GNG7-FLAG*, RNAi-resistant mutant cells, U2OS, and U2OS-*GNG7-GF* for immunofluorescence were grown on coverslips in 24-well plates with or without RNAi. Cells were washed once with PBS and fixed by −20°C methanol for 5 minutes or 4% formaldehyde at room temperature for 20 minutes, and then washed with TBS-Tx (TBS supplemented with 0.1% Triton X-100) and blocked by AbDil-Tx (TBS-Tx with 2% BSA and 0.05% sodium azide) at room temperature for 30 minutes. Covers were stained with primary antibodies and incubated at 4°C overnight, followed by probed with the secondary fluorescently conjugated antibodies at room temperature for 1 hour. To visualize cytoskeleton, F-actin fibers were directly stained with phalloidin (Cell Signaling). After rinsed thoroughly by TBS-Tx, covers were mounted in anti-fade prolong Gold with DAPI. The primary antibodies used in immunofluorescence experiments were as follows: GFP (abcam), FLAG (sigma), SQSTM1/p62 (Cell Signaling), LC3B (Cell Signaling), and Tubulin (TransGen Biotech). Images were taken using a Leica MI4000B fluorescent microscope. All experiments were repeated at least three times and representative micrographs are shown in the Figures.

### Western blotting

The expression levels of GNG7 and various cellular proteins related to cell cycle and autophagy were determined using Western blotting assay. Cells were lysed on ice with the M-PER lysis buffer supplemented with protease and phosphatase inhibitor cocktail (Roche) at 4°C for 20 minutes. Protein samples were electrophoresed on 12% or 15% SDS-PAGE gels after thermal denaturation at 95°C for 8 minutes with 2×SDS loading buffer (20 mM Tris-HCl pH 8.0, 100 mM DTT, 2% SDS, 20% Glycerol, and 0.016% Bromophenol Blue), and transferred onto the PVDF membranes (Millipore) by Thermo Scientific Owl VEP-2. The membranes were blocked with 5% no-fat dried milk at room temperature for 30 minutes and followed by incubating them with corresponding primary antibodies, including phospho-MTOR (Ser2448), MTOR, RAPTOR, RICTOR, GβL, SQSTM1/p62, LC3B, phospho-S6K (T389/412), S6K (all 1:1000 dilution, Cell Signaling), and GNG7 (1:200, abcam), CCNB (1:100, Santa Cruz), FLAG (sigma), α-Tubulin, and β-Actin (1:1000, TransGen Biotech). HRP-linked secondary anti-rabbit or anti-mouse antibodies (1:5000, Cell Signaling) were then incubated at room temperature for 1 hour. Visualization was performed using enhanced chemiluminescence Kits (ECL, Millipore or Thermo Scientific) and the blots were analyzed using Tanon Fine-do X6 (Tanon). Protein levels were normalized to the matching densitometric value of the internal control α-Tubulin by ImageJ software.

### Co-immunoprecipitation

HeLa-*GF* and HeLa*-GNG7-GF* cells were cultured in 150 mm dishes to 95–100% confluence and lysed as above. The whole cell lysate was centrifuged at 4°C, 13,000 g for 10 minutes and the supernatants were mixed with FLAG antibody pre-conjugated Dynabeads protein G (Invitrogen) at 4°C for 2 hours on a rotator. After washing with ice-cold-PBST (PBS with 0.02% Tween-20) three times (every 5 minutes per time) at 4°C on a rotator, the Dynabeads were collected using the magnet. And the Dynabeads-FLAG-protein mixture was supplemented with lysis buffer and 2×SDS loading buffer followed by boiling at 95°C for 8 minutes. The cell lysate after centrifuging was used as input control. Western blotting was carried out to analyze the co-immunoprecipitation results.

### Cell synchronization assay

HeLa and HeLa-*GNG7-GF* cell lines were seeded at a concentration of 1 × 10^5^ cells/cm^2^ in 35 mm dishes and synchronized by double thymidine method. DMEM with 2.5 mM thymidine was added to cells at 30% confluence for 16 hours. After washed three times with PBS to remove thymidine, cells were incubated with fresh DMEM medium for 8 hours. Then DMEM medium containing 2.5 mM thymidine was added for another 16 hours for the 2nd thymidine block to reserve cells at G1/S transition. “0” indicated the beginning of double thymidine release. Synchronized cells were collected at needed time points after released for western blotting or RT-PCR.

### Annexin V/PI apoptosis detection assay

HeLa cells were plated in 6-well plates at 1 × 10^6^ cells/cm^2^. After 12 hours, cells were transfected with 0, 0.05, 0.1, 0.25, 0.5 and 1.0 μg/ml *GNG7-FLAG* plasmids respectively, or treated with 500 nM staurosporine for 24 hours. All cells were collected 48 hours later and washed twice with ice cold PBS. FITC-Annexin V apoptosis detection kit (BD PharmingenTM) was used in this assay. Cells were then resuspended in 1× binding buffer at a final concentration of 1 × 10^6^ cells/ml before transferred to 5 mL culture tube. FITC-Annexin V (5 μL) and PI (5 μL) were added to 100 μL cell solution, mixed and incubated in the dark for 15 minutes at room temperature. Then, 400 μL of 1× binding buffer was added to the stained cells before they were analyzed by flow cytometry within 1 hour. Data were analyzed using FlowJo.

### Cell cycle analysis

Transfected HeLa cells were trypsinized and washed with PBS before they were fixed in 70% ice-cold ethanol overnight at 4°C. Then they were washed again with PBS and incubated in PI solution (BD Pharmingen) for 30 minutes at room temperature. The samples were examined by flow cytometry on a BD Flow Cytometry (BD Bioscience). 1 × 10^4^ cells per sample were collected for each condition. Data were analyzed using ModFit LT.

### Cell growth curve

HeLa, HeLa-*GNG7-GF*, U2OS, and U2OS-*GNG7-GF* cell lines were seeded at 2 × 10^5^ cells/cm^2^ in 30 mm dishes and taken photos 24 hours after planting. The pictures were taken at least 8/cell for cell numbers analyzing at 24 hours intervals for 7 days each time. Using formula to calculate the cell growth doubling time: ${\text{T}}_{\text{D}}=\text{t}\frac{\log2}{\log\;{\text{N}}_{\text{t}}-\log\;{\text{N}}_{0}}$. In this formula, t is incubation time; N_0_ is first counting cell numbers; Nt is cell numbers after incubation time. In this experiment, N_0_ and Nt are both relative cell numbers. Experiments were repeated for at least three times.

### Statistical analysis

All images shown in figures are representatives of repeated experiments. For quantifications, mean values are shown in the figures, and Standard Deviations or Standard Error of Means are shown as error bars. Comparisons between treatments were analyzed by a two-tailed Student *t* test or by one-way ANOVA. *P* values are labeled in the figures for where data were compared.

## SUPPLEMENTARY MATERIALS FIGURES


