# Supplementary material for: G protein γ subunit 7 induces autophagy and inhibits cell division
Source: Oncotarget. 2016 Apr 2;7(17):24832–47. doi: 10.18632/oncotarget.8559 (PMC5029746; doi:10.18632/oncotarget.8559)
Supplement: Supplementary file 1 [file oncotarget-07-24832-s001.pdf]

G protein γ subunit 7 induces autophagy and inhibits cell division

Supplementary Materials

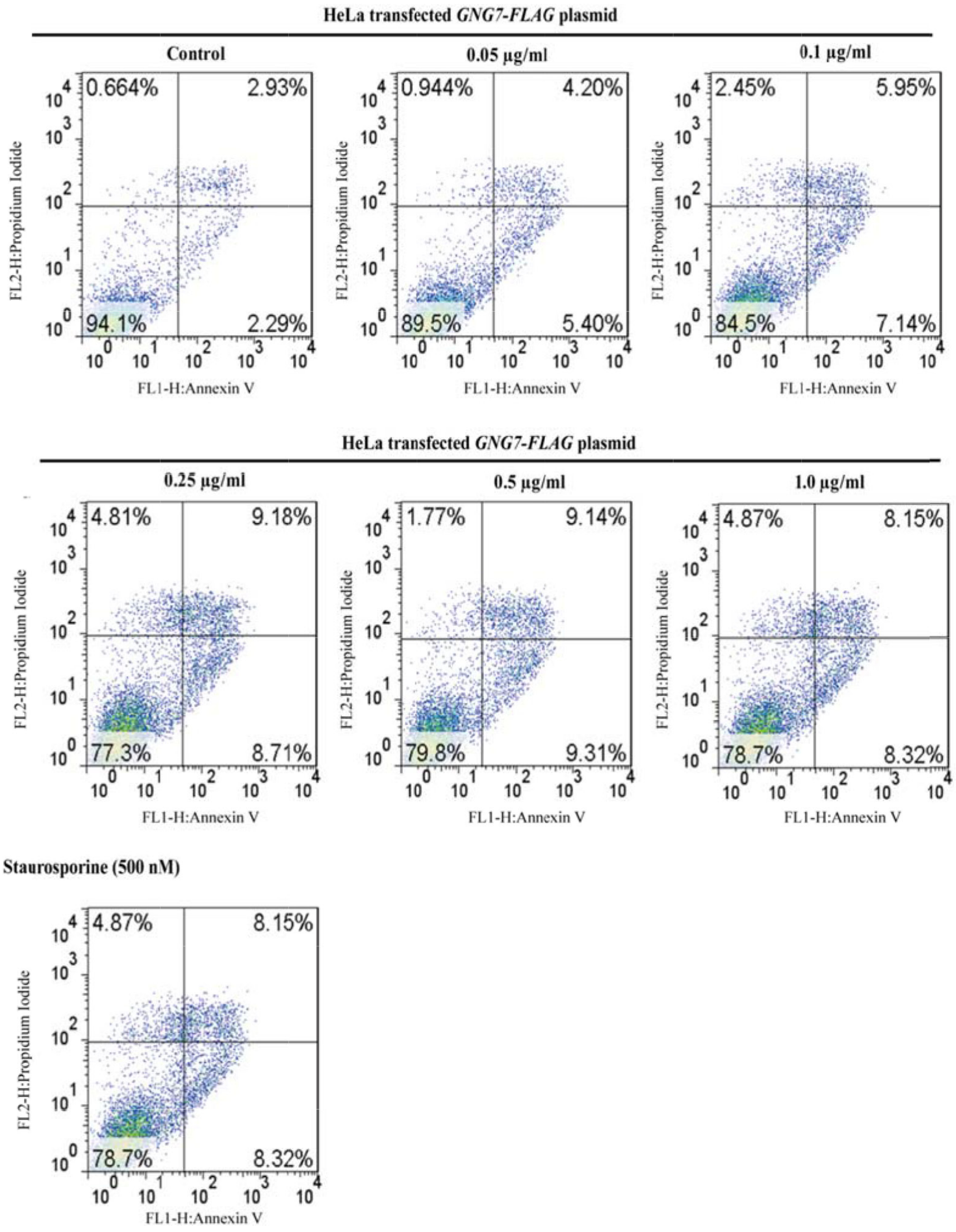

Supplementary Figure S1: GNG7 overexpression causes cell death. HeLa cells were transfected with 0, 0.05, 0.1, 0.25, 0.5 and 1.0 μg/ml *GNG7-FLAG* plasmid for 48 hours or treated with 500 nM staurosporine for 24 hours and then analyzed using Annexin/PI assays. Apoptotic and dead cells were increased in a dose-dependent manner.

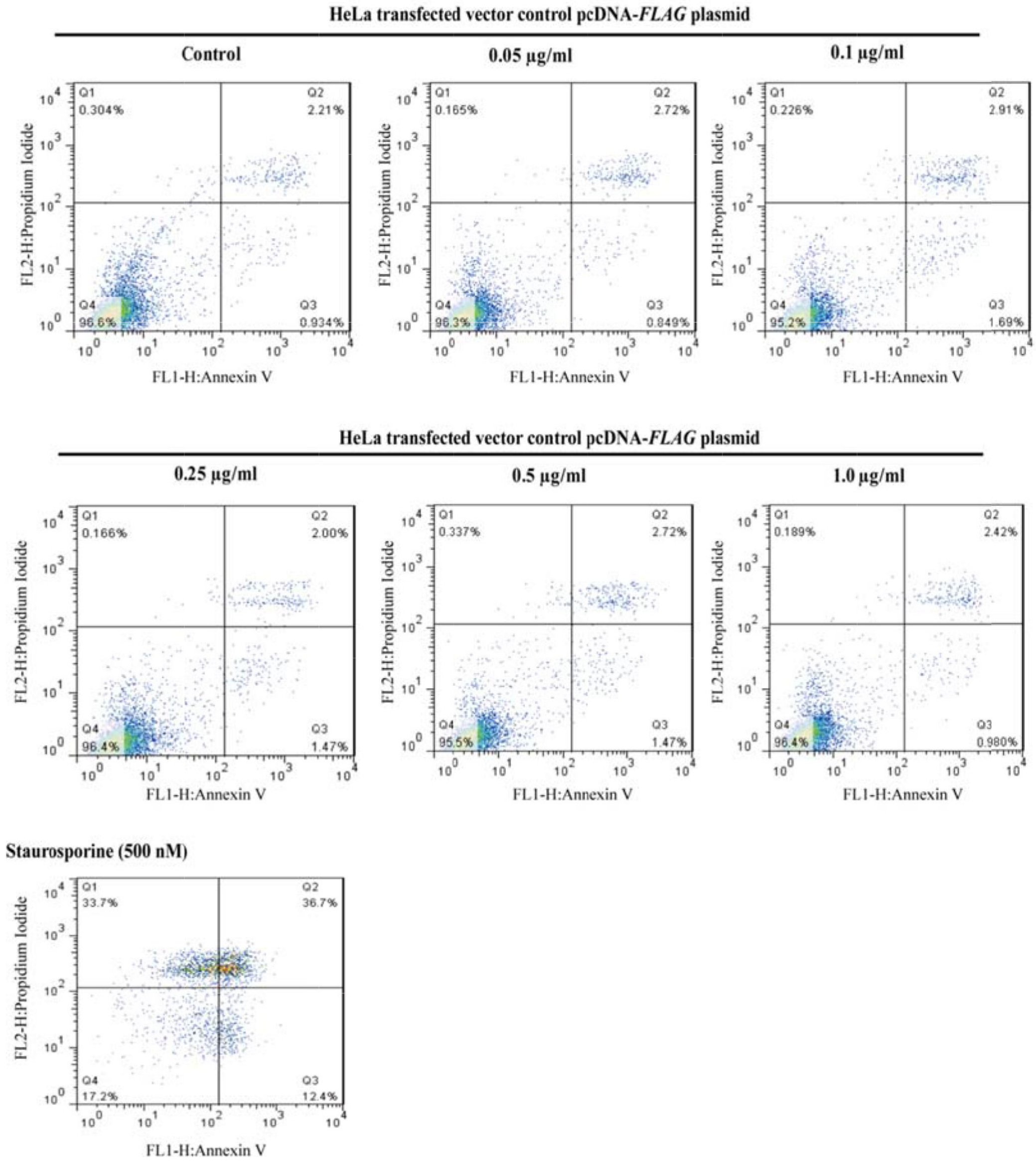

Supplementary Figure S2: Vector control overexpression does not cause cell death. HeLa cells were transfected with 0, 0.05, 0.1, 0.25, 0.5 and 1.0 µg/ml vector control pcDNA-*FLAG* plasmid for 48 hours or treated with 500 nM staurosporine for 24 hours and then analyzed using Annexin/PI assays. Apoptotic and dead cells did not increase.

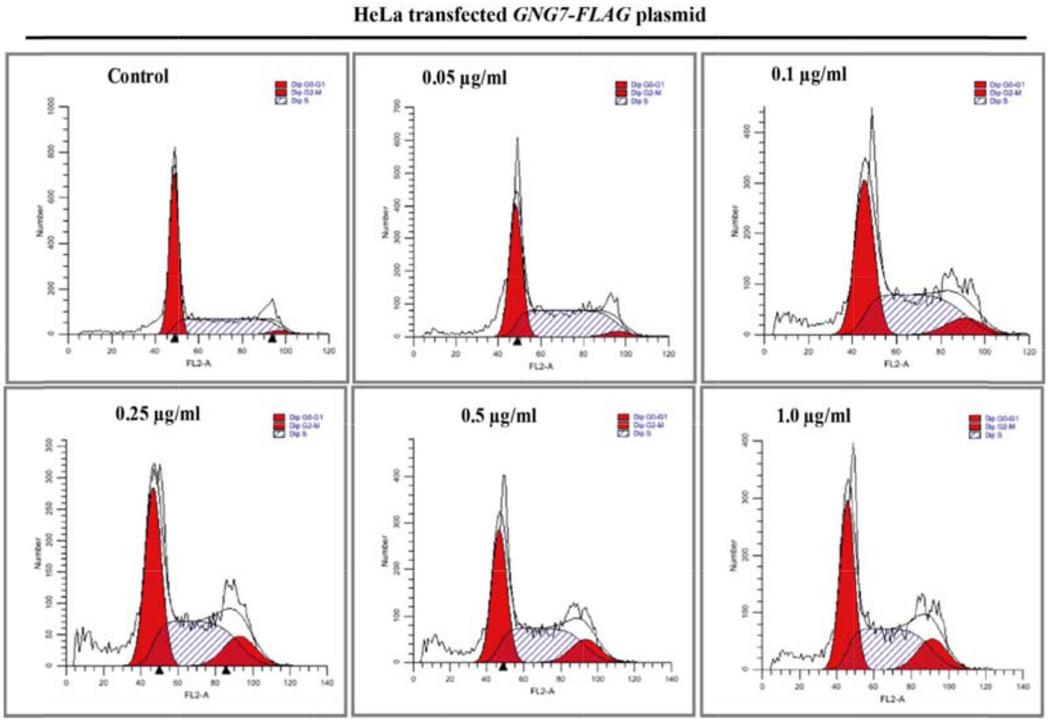

Supplementary Figure S3: GNG7 overexpression arrests cells in M phase. HeLa cells transfected 0, 0.05, 0.1, 0.25, 0.5 and 1.0 µg/ml *GNG7-FLAG* plasmid were collected for flow cytometry assay to show cell cycle distribution. Quantification results showed that G2-M cells were increased in a dose-dependent manner.

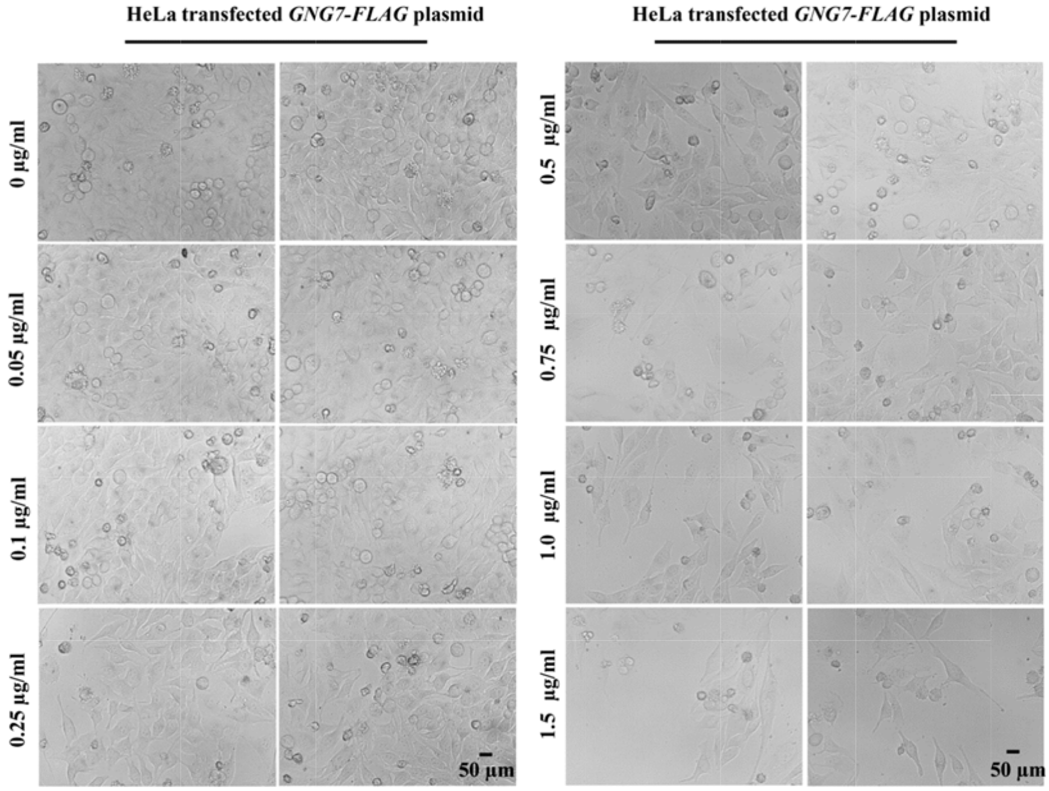

Supplementary Figure S4: GNG7 overexpression has no influence on cell senescence. HeLa cells were transfected with 0, 0.05, 0.1, 0.25, 0.5, 0.75, 1.0 and 1.5 µg/ml *GNG7-FLAG* plasmid for 48 hours. Senescence assay was carried out according to instructions of the senescence β-Galactosidase Staining Kit.

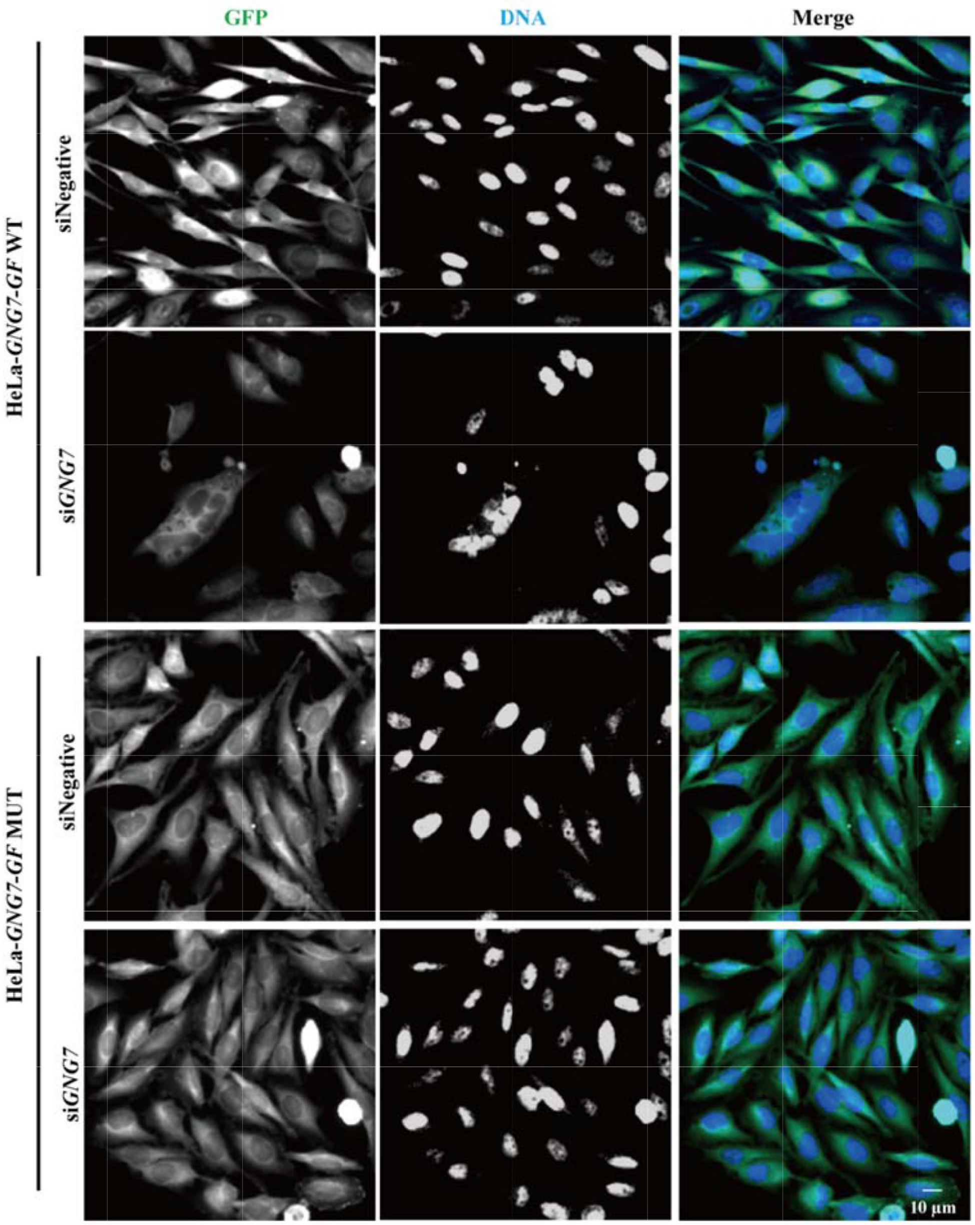

Supplementary Figure S5: GNG7 knockdown causes bi/multinucleated cells in HeLa-*GNG7-GF* WT cells but not the RNAi resistant mutant HeLa-*GNG7-GF* MUT cells. HeLa-*GNG7-GF* WT and MUT stable cell lines were knocked down with negative or GNG7 siRNAs for 72 hours. GFP and DNA were stained. *GNG7* could be mostly knockdown in WT, correspondently with an increased percentage of bi/multinucleated cells. In MUT cells si*GNG7* treatment had no change compared to negative shown by GFP staining.

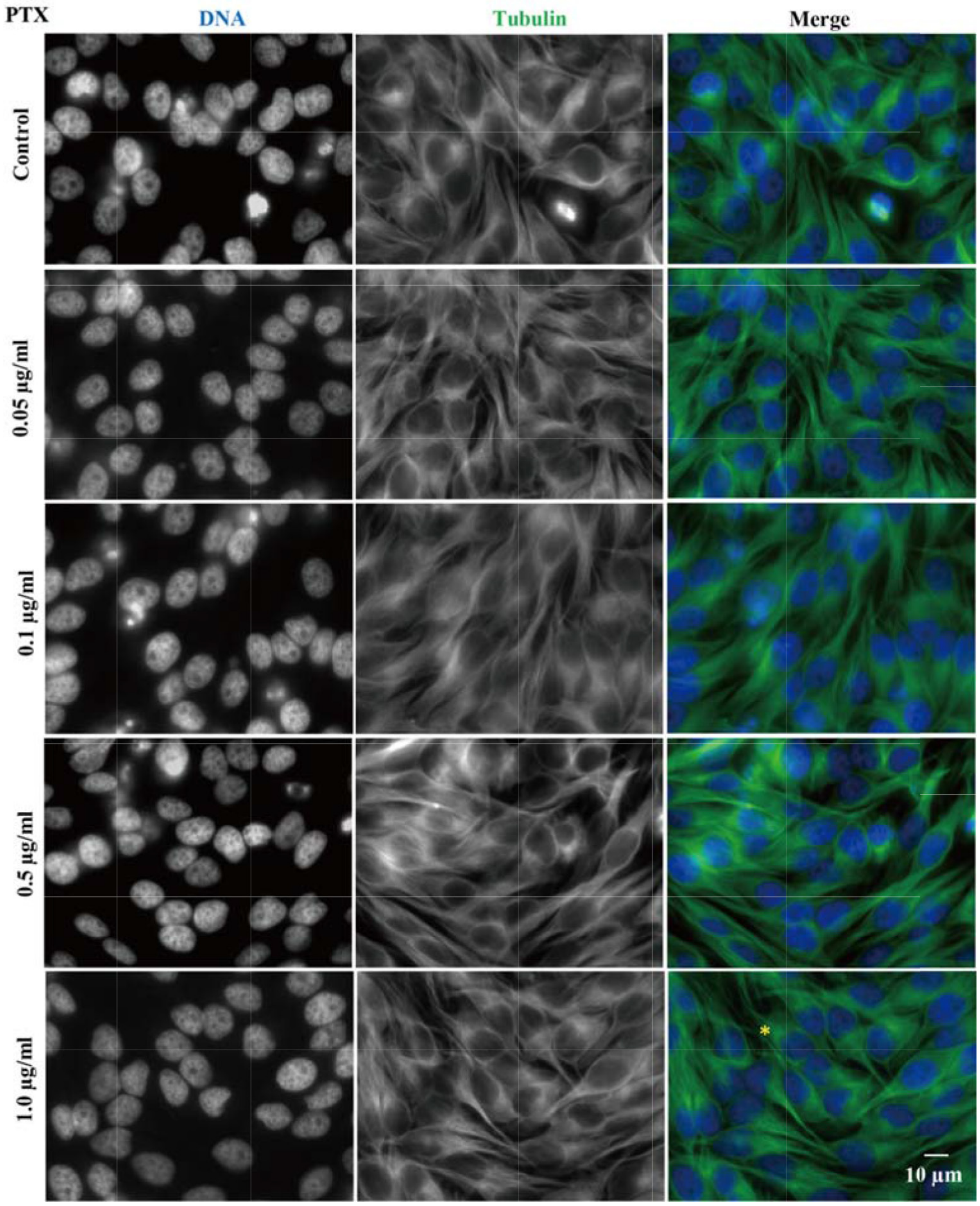

Supplementary Figure S6: PTX does not induce bi/multinucleated cells significantly. HeLa cells were plated 12 hours before adding 0, 0.05, 0.1, 0.5 and 1.0 µg/ml pertussis toxin (PTX). 72 hours later, cells were stained with tubulin and DNA. There were only a few binucleated cells after 0.5 and 1.0 µg/ml PTX treatment.

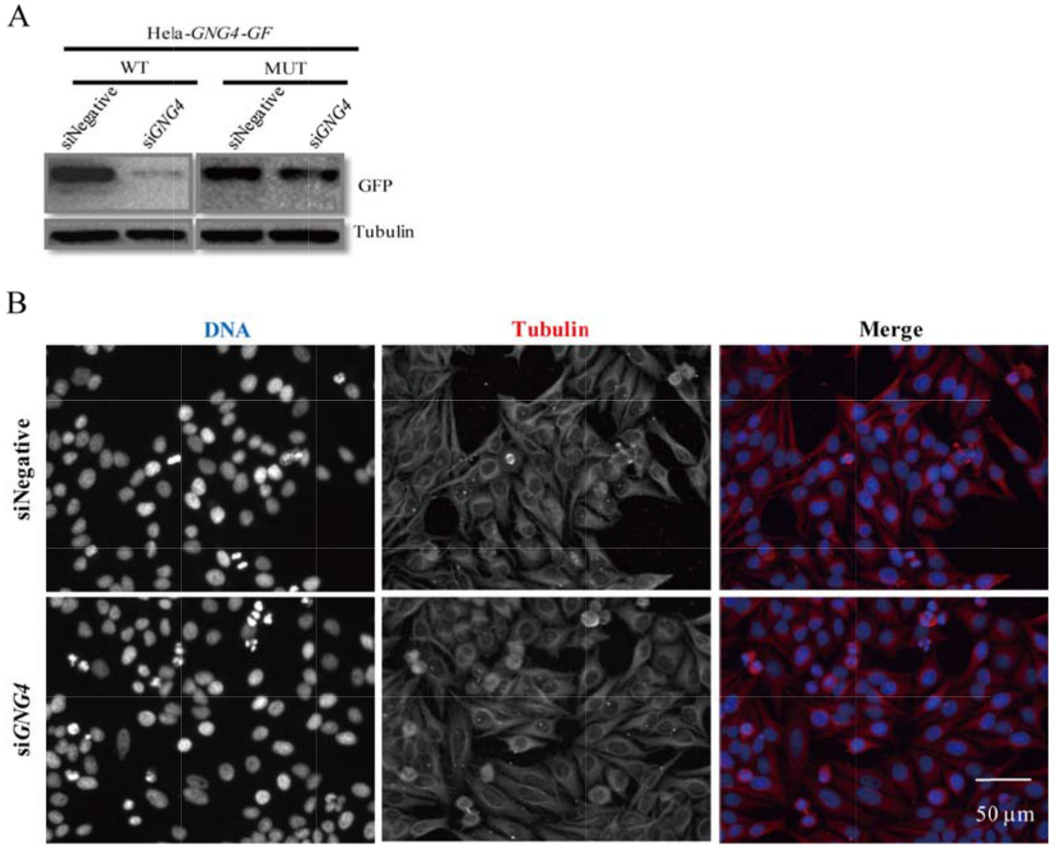

Supplementary Figure S7: GNG4 RNAi does not induce binucleated cell formation. (**A**) *GNG4* siRNA specifically knockdown GNG4. HeLa, HeLa-*GNG4-GF*-WT and HeLa-GNG4-GF-MUT (RNAi resistant mutant) cells were transfected with control or *GNG4* siRNAs for 72 hours before they were lysed analyzed for Western blots. (**B**) GNG4 RNAi does not induce bi/multinucleated cells. HeLa cells were transfected with siRNAs for control or *GNG4* for 72 hours before they were fixed and stained for microtubules and DNA with anti-tubulin antibody (Red), and DAPI (blue).
